# Feeding the future global population

**DOI:** 10.1038/s41467-023-44588-y

**Published:** 2024-01-03

**Keywords:** Agroecology, Agricultural genetics, Sustainability

## Abstract

Climate change is exacerbating challenges both for global food production and from its environmental impacts. Sustainable and socially responsible solutions for future world-wide food security are urgently needed.

We continue our series of collections on the Sustainable Development Goals (SDGs) with a Collection and call for papers focused on Sustainable Food Production. Food production connects several of the 17 SDGs given its fundamental—and frequently opposing—impacts on human populations and the environment. Adequate food production to ensure food and nutritional security is at the center of SDG Goal 2: Zero hunger. However, food production also underlies a significant portion of the human impact on climate and the biosphere. Furthermore, these impacts often occur in regions distant from where the food is actually consumed and thus potentially increase global inequalities. Therefore, sustainable increases to food production must accommodate protection of the environment and biodiversity (Responsible consumption and production, Climate action, Life below water and Life on land: Goals 12–15) in order to be successful in the overarching goal of reducing societal inequalities (No poverty, Good health and wellbeing, Reduced inequalities, and Peace, justice and strong institutions: Goals 1,3,10, and 16).

Achieving global food security sustainably will not be easy given that the United Nations (UN) estimates the human population will reach 8.5 billion in 2030 and 9.7 billion in 2050. While the overall population growth rate is estimated to be slowing, it is nonetheless projected to remain high in several regions of Africa and Asia, including areas already at risk of food insecurity. Meanwhile, in affluent countries, there are substantial inequalities in access to nutritious food despite high levels of food waste overall.

Historical increases in food production have been driven in large part by land use change, which is a major contributor to biodiversity loss and can play a role in climate change. However, scientific innovation can also boost productivity. The Green Revolution of the mid-20^th^ century was crucial in improving food security, in part through the use of high-yielding crop varieties. Since then, advances in plant breeding have also been key to strengthening the reliability of food production systems. Yet, crop varieties bred for current climate conditions may not continue to deliver high yields when confronted with a rapidly changing environment. Continued scientific innovations will therefore be necessary to enhance the resilience of food production infrastructures and management systems, and to inform strategies such as modifying sowing calendars to take climatic change into consideration^1^.

The environmental impact of food consumption can also be confronted by making diets more sustainable. For example, the EAT–*Lancet* Commission proposed a universal reference diet known as the ‘planetary health diet’, aiming to improve health without surpassing planetary boundaries^2^. Transition towards more sustainable diets may nonetheless prove difficult. Terrestrial animal farming emits more carbon than plant-based meat and dairy alternatives (in addition to having a large impact on land use), while the latter two entail significant water footprints and natural ecosystem loss due to crop expansion^3^. Alternative food sources such as cultured meat and insect-based products are increasingly accepted by consumers, but their impact on the environment and human health remains to be firmly established^3^. Blue foods—aquatic foods captured or cultivated in marine and freshwater systems—are also receiving increased attention given their high nutritional value, but their sustainability is contested^4^.

Economic barriers to a sustainable and healthy diet also need to be considered in solving food and nutritional insecurity within planetary boundaries. Substituting animal products with plant-based or future foods (seaweed, cultured meat, or insect foods) might reduce food prices in upper-middle-income countries but increase costs in lower-middle income regions^5^. It is also estimated that global cropland expansion and intensification could disproportionately affect biodiversity in the Global South, while the Global North would benefit from an increase in global food production and reduced market prices with limited risk to their local ecosystems^6^. Social justice principles should guide the design and implementation of food security solutions, so that deeply rooted social inequalities are alleviated rather than exacerbated by global changes in diet and increased agricultural output.

It is clear that we must not ignore the environmental and socio-economic issues associated with current food production and consumption systems, and that science must play a significant role in finding solutions. The articles in our Sustainable Food Production collection highlight some of the challenges and recent progress made in the areas of food security and sustainable agriculture that reflect *Nature Communications*’ commitment to sustainability and social justice.
